# The impacts of delivery mode on infant’s oral microflora

**DOI:** 10.1038/s41598-018-30397-7

**Published:** 2018-08-09

**Authors:** Hongping Li, Jun Wang, Lijuan Wu, Jun Luo, Xia Liang, Bin Xiao, Yuanfang Zhu

**Affiliations:** Bao’an Maternity and Child Health Hospital, Shenzhen, 518000 China

## Abstract

This study investigated the effects of different delivery modes on oral microflora in healthy newborns immediately post-partum, and provided evidence for microbial colonization disruption induced by medical procedures. Eighteen infants delivered by cesarean section and 74 by vaginal delivery were included in the study. High-throughput sequencing of 16S bacterial rRNA was performed on oral samples collected immediately after birth. All data were analyzed using bioinformatics approaches. Our results indicated that different oral bacteria were found between infants delivered by cesarean section compared to vaginal delivery group. Lactobacillus, Prevotella and Gardnerella were the most abundant genera in the vaginal group, while Petrimonas, Bacteroides, Desulfovibrio, Pseudomonas, Staphylococcus, Tepidmicrobium, VadinCA02, and Bifidobacterium were dominant bacteria in the cesarean section (C-section) group. Furthermore, bacteria isolated from 27 vaginally-delivered infants were not clustered into the vaginal group. Most of them spent more than 24 hours in the delivery room and this led to repeated sterilization procedures. We hypothesized that repeated sterilization might have influenced oral microflora in those cases. To conclude, this study suggested that different modes of birth delivery affect oral microflora in healthy infants. In addition, attention shall be paid to the clinical practice of repeated sterilization of the vulva that possibly obstructs the colonization of vaginal bacterial.

## Introduction

The gut bacterial colonization and evolution begins at birth, and consequently plays an important role in infants’ growth development, nutrition, metabolism and immunization^1–4^. The gut bacteria are also strongly associated with human health (e. g. associations between gut microbiota and gastrointestinal diseases) by controlling the homeostasis of the gut microbiota^5^. It has been identified that the delivery mode is a significant factor influences the colonization and composition of the intestinal microbiota^6^. Compared with the infants delivered by *vaginal delivery*, those by cesarean section *(C-section)* have less diverse intestinal microbiome and are more likely to develop diseases such as asthma, obesity and diabetes^7^. Nevertheless, it has been reported that some vaginally delivered infants lack *Bifidobacteria* in their guts^8^, suggesting that other factors may also interfere the intestinal microbiota.

Newborn acquires mother’s microbiome from birth canal during the delivery process and these bacteria then colonize in the gut. Studying the oral microbiome of infants offer a good perspective for us to understand the gut microbiome disruption caused by C-section. A previous study of three-month-old infants reported higher amount of bacterial taxa of the oral microbiota in vaginally delivered infants than the ones with other birth modes^9^. Pacifier use and other feeding modes may also affect infant’s oral microbiota composition. Therefore, the timing of the sample collection is critical. In this study, we collected the samples immediately after birth, and we compared the differences in colonization patterns of the oral microbiota between the infants born via different delivery modes to study the possible factors that may affect the infants oral microflora.

## Results

A total of 100 participants were recruited, including 80 vaginal births and 20 C-section cases. Among the participants, 2 C-section and 6 vaginal delivery babies did not meet the inclusion criteria. 92 participants were included in the final study and were divided into two groups: vaginal delivery group and C-section group. Continuous variables are compared by Student’s independent t-test and categorical variables are compared using Chi square test and Fisher’s exact test. Statistical significance is defined as p < 0.05. No differences in mother’s age, gestation week, pregnancy weight gain, gestational diabetes, infant sex or infant’s birthweight were found between the two groups (Table 1).

**Table 1:** Clinical characteristic of mother and infant from both the c-section and the vaginal delivery groups.

Variables	Vaginal delivery group N = 74	C-section group N = 18	P value
Mother’s age	27.9 ± 3.6	28.4 ± 4.1	0.63
Number of male infants	35	11	0.43
Gestational week	39.5 ± 1.2	39.6 ± 1.3	0.79
Birth weight (gram)	3 183 ± 356	3 326 ± 50	0.29
Gestational weight gain (kilogram)	13.2 ± 4.1	14.9 ± 3.2	0.07
Number of Gestational diabetes mellitus (GDM)	9	2	0.99

Continuous variables were presented as mean ± standard deviation, while group comparisons were conducted by independent T test. Categorical variables were presented as count, and relevant group comparisons were performed using Chi-square test or Fisher Exact test. *P* < *0.05* was defined as statistically significant.

**Figure 1:**
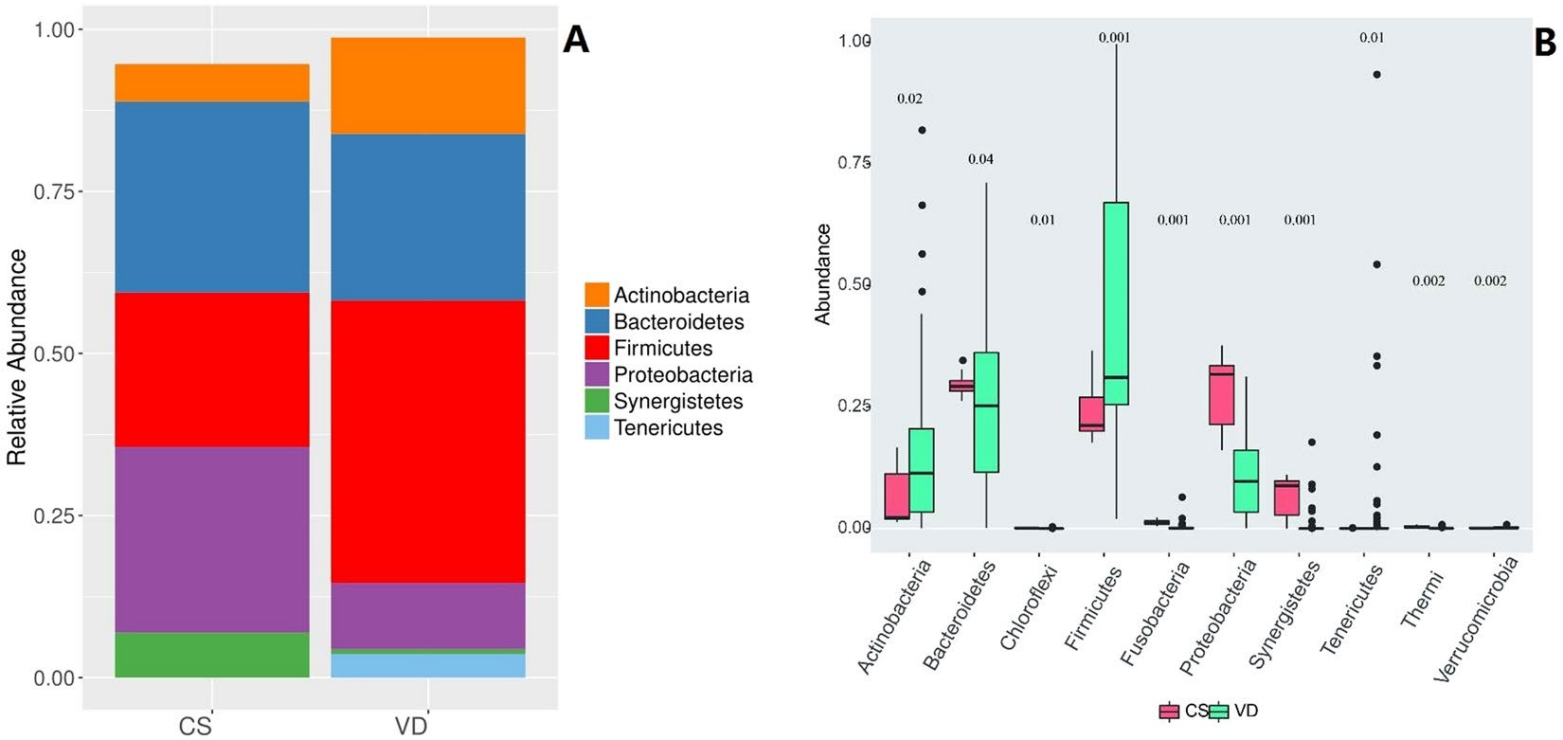
Oral bacterial phyla in infants delivered by cesarean section compared to those born by vaginal delivery. CS stands for C-section and Vd stands for vaginal delivery. (**A**) Relative abundance of bacteria analyzed by LEfSe software. (**B**) Significant difference in bacterial phyla between the vaginal delivery group and the C-section group. P values for the comparison across the two groups are placed above the boxes plot.

According to relative abundance of the bacterial, mainly six phyla of the oral bacteria, were identified using LEfSe software^10^. They are Actinobacteria, Bacteroidetes, Firmicutes, Proteobacteria, Synergistetes and Tenericutes (Fig. 1A). Within the vaginal delivery group, the most abundant phylum are Firmicutes, Bacteroidetes and Actinobacteria. For the C-section group, the most abundant phylum are Bacteroidetes, Proteobacteria and Firmicutes. Comparison at phylum level of oral bacterial across the two groups was made. Method employed here is Metastats^11^. There were significantly more Actinobacteria, Firmicutes and less Bacteroidetes and Proteobacteria, in the vaginal delivery group than the C-section group (Fig. 1B).

**Figure 2:**
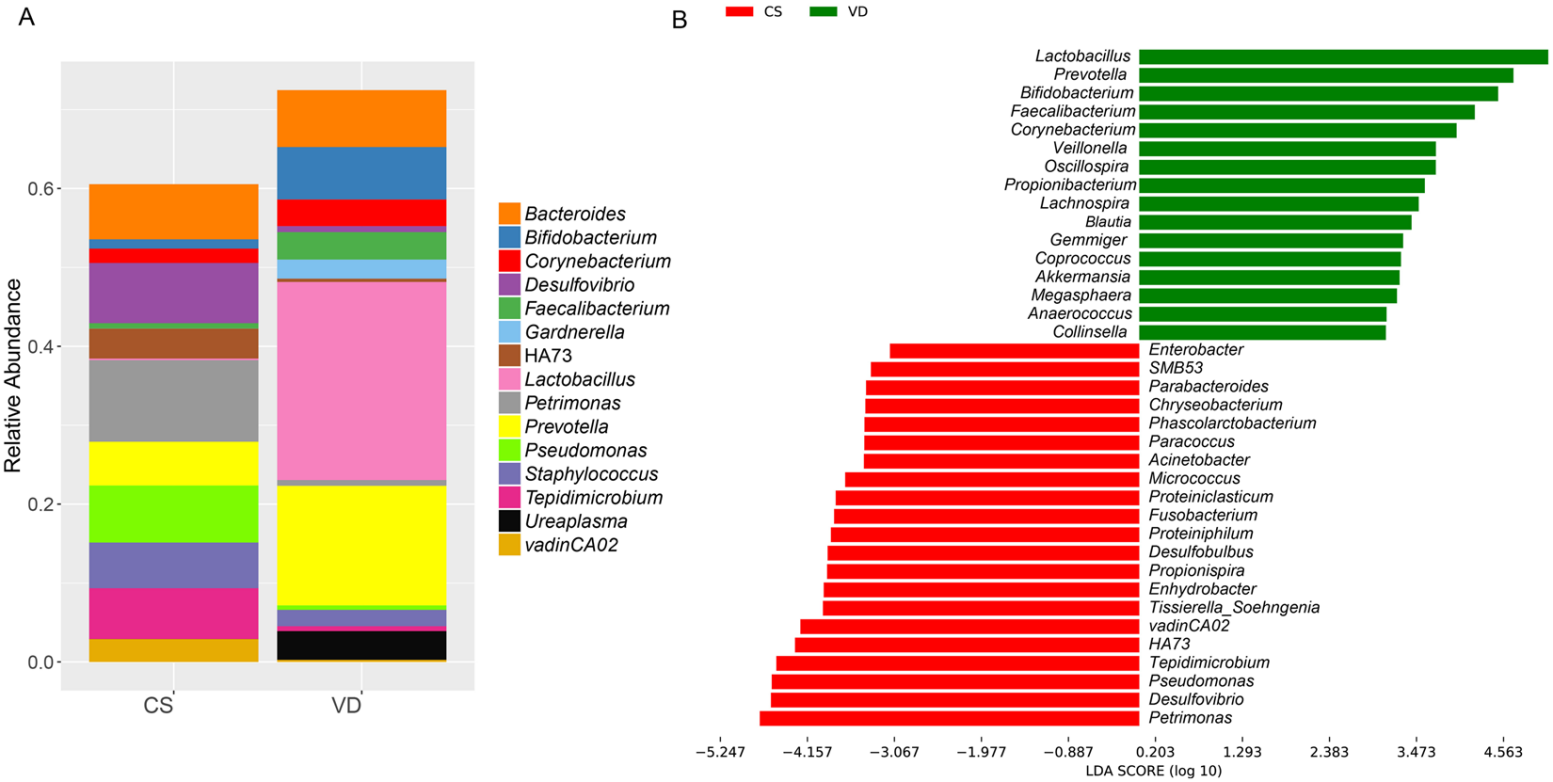
Oral bacterial genus in infants delivered by cesarean section compared to those born by vaginal delivery. VD stands for vaginal delivery group and CS stands for C-section group. (**A**) Relative abundance of bacteria analyzed by LEfSe software. (**B**) LEfSe comparison of oral microbiomes from infants of the two groups. Bacterial genus in samples isolated from vaginally delivered infants with a positive linear discriminant analysis (LDA) score are in green; C-section samples with a negative LDA score are shown in red (differences at the level of 0.1%).

At the genus level, we made similar analysis. Within the vaginally-delivered group, *Lactobacillus, Prevotella, Bifidobacterium, Corynebacterium, Bacteroides, Gardnerella*, and *Ureaplasma* were the dominant bacterial genera; while *Petrimonas, Bacteroides, Desulfovibrio, Pseudomonas, Staphylococcus, Tepidmicrobium, VadinCA02* and *Bifidobacterium* were the most common genera in the C-section group (Fig. 2A). With the exception of *Bacteroides*, significant differences in bacterial abundance were observed between the two groups (Fig. 2B). *Lactobacillus* and *Petrimonas* are the two genera vastly different across the two groups.

**Figure 3:**
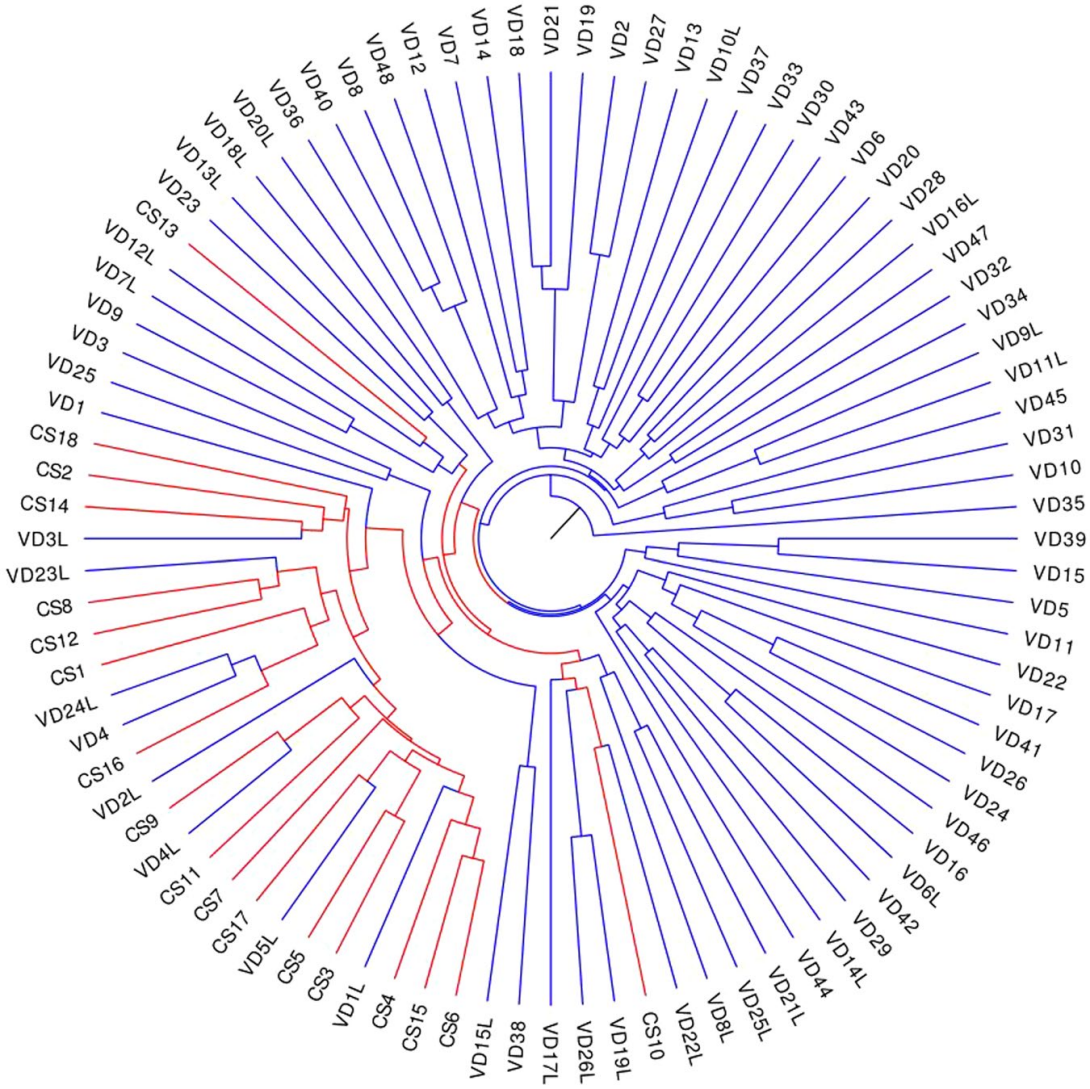
Unweighted unifrac clustering analyses between the two studied groups. Blue indicates vaginal delivery and red represents C-section. Each participant to be clustered was assigned a label in the figure. For instance the CS2 label means the second mother in the C-section group while VD4 means the fourth mother in the vaginal delivery group. VD1L represents the first of those mothers who both have a vaginal delivery and spent relatively more time in the delivery process.

Unweighted unifrac clustering demonstrated that the overall vaginal birth group and C-section group were separated. All 18 C-section cases were perfectly clustered into one group, while 27 vaginal delivery cases were clustered with the C-section group. We searched the medical records and clinical history of these 27 patients and found that most of them having longer time in the delivery process. We compared their time spent in the delivery room to that of the rest C-section cases and found a significant difference (27 cases: 2532 min ± 1580, the rest: 581 min ± 443, P < 0.001). Also, 8 cases with emergent vaginal delivery were all correctly clustered into the vaginal group (Fig. 3).

## Discussion

Several studies have found that infants born by C-section have a higher risk of developing allergic rhinitis, asthma, eczema, obesity and diabetes when comparing with infants born vaginally^12,13^. Some other studies have also suggested possible links between the aforementioned allergic diseases and gut microflora^4,14^ that affected by mode of delivery. A study by Dominguez (2010) has shown that bacteria from multiple body habitats in naturally delivered newborns were similar to mother’s vaginal bacteria, while microbial communities in newborns delivered by C-section resembled that of the mother’s skin^15^. In this study, we collected infant’s oral samples immediately after birth, before they had any contact with mother’s skin. Oral samples were immediately obtained after birth. Our data demonstrated that differences between groups in oral microflora can be attributed to the vaginal contact. The results from this study help to elucidate the microbial environment of infants born by different delivery modes.

The fact that oral cavities of newborns from the C-section group harbored a large variety of bacteria, especially dominanted by the anaerobic *Bifidobacteria* that is by no means airborne, suggested that the acquisition of bacteria occurred within the uterus. Thirty cases from the natural labor group had *Lactobacilli* as the dominant bacteria, which is consistent with the normal composition of vaginal microflora. Normally *Lactobacilli* is dominant in vagina and it protects the integrity of intestinal mucosa^16^. Both *Bifidobacteria* and *Akkermansia* are beneficial to human’s health. Abundance of the former in gut helps to maintain balance of immune system and reduce allergic diseases^17^. The latter protect us against metabolic conditions such as obesity and diabetes^18^. In our study the vaginal delivery group has significantly more of these two bacterial, providing evidence of the healthy aspect of natural delivery. Two common pathogenic bacteria, *Providencia* or *Gardnerella*, was also found in 15 naturally delivered infants. The presence of these bacteria could be explained by the inclusion of mothers with unmanifested vaginal infection and we believe this still conforms to the regular microbiota pattern within vaginal cases. All cases from the C-section group clustered together in the clustering analysis, while the vaginal delivery group was not consistent. Among all the vaginally delivered cases, we found 27 cases that were categorized into the C-section group following clustering analysis. Clinical records suggested that within these 27 cases, most mothers spent a longer amount of time in the delivery room and underwent more pelvic exams. These results suggested that not all vaginally delivered infants could acquire their mother’s vaginal microflora. In 2010, a WHO survey reported that C-section rate in China has reached 46.2%, of which 11.7% were with no indications^19^. The hospital in which this study was conducted, managed to reduce the C-section rate to 28%. Nevertheless, according to the obtained results, there were still 36.5% (27/74) of infants who could not benefit from vaginal delivery in terms of gut microflora colonization. In her study, Dominguez swabbed the C-section infant’s multiple body parts with gauze that was placed in the mother’s vagina for 1 h before C-section, and observed partial microbial restoration as these C-section infants were more likely to be clustered with vaginally delivered cases^20^.

We noticed that women who spend more time in the delivery room received repetitive routine pelvic exams (one exam every two hours) and these procedures were associated with the administration of a large amount of povidone iodine. The exposure to iodine through aforementioned procedures, and through contact with doctor’s gloves, inevitably led to the spread of iodine into patient’s vagina. It can be hypothesized that iodine might have affected the vaginal microbiota by preventing the transfer from mother to infants. Our hypothesis can furthermore be cross-validated by the fact that 8 emergent delivery cases, less likely to undergo pelvic exams, were correctly clustered as vaginal delivery.

According to one highly referenced Chinese textbook of obstetrics and gynecology commonly used in medical colleges, it is necessary to cleanse the vulva using soap and water, while povidone iodine should be used for sterilization purposes prior to pelvic exam^21^. To the best of our knowledge, lots of Chinese hospitals strictly adhere to this principle. Our study suggests that sterilization procedures may prevent infants from obtaining their mother’s vaginal microflora, indispensable for infant’s health. Further studies are necessary to examine the necessity of the sterilization procedures and its impact upon infants microbiota and health.

## Methods

All procedures and experiments in this study were approved by the Bao’an Maternity and Child Health Hospital’s ethics committee. Experiments were performed in accordance to relevant guidelines and regulations. Written informed consent was obtained from each participants in accordance to Bao’an Maternity and Child Health Hospital’s guidelines.

### Inclusion criteria

Pregnant women who gave birth to healthy newborns at the Shenzhen Bao’an Maternal and Children’s Hospital during the period from October to December 2016, were recruited to participate in this study. The inclusion criteria were: gestational age <37 weeks, birth weight >2500 g; maternal pre-pregnancy body mass index (BMI) 19–25, and normal antenatal test results. Exclusion criteria were: birth weight <2500 g, deformity, asphyxia or unstable respiratory conditions, mothers with chronic metabolic disease or severe infectious disease history, or intake of antibiotics for more than 3 consecutive days. Participants were categorized into vaginal delivery group and C-section group according to information from their delivery records.

### Sample sequencing

Bacterial DNA was analyzed using 16S rRNA V3-V4 sequencing methods. Oral samples were obtained by oral buccal swab immediately after birth, subsequently transferred in sterile bags and stored at −80 °C until further processing.

Bacterial DNAs was isolated using PowerSoil® DNA Isolation Kit according to manufacturer’s instructions. Bacterial DNA was then purified by gel electrophoresis (0.7% agarose-gel) followed by phenol extraction^22^ and elution. DNA quality was measured according to the absorbance ratios of 260/280 nm and 260/230 nm using a NanoDrop ND-1000 Spectrophotometer (NanoDrop Technologies, Wilmington, DE, USA), and was consequently quantified using Qubit dsDNA HS Assay kit (Life Technologies). The Polymerase Chain Reaction (PCR) reaction was performed on a 96-well Thermal Cycler (Life Technologies). The amplicon libraries were generated using a high-fidelity polymerase (Invitrogen), and then purified using AMPure XT beads. PCR amplification was carried out by adding index 1 (i7), index 2 (i5), and 2 adaptors (P5 and P7) for cluster generation and sequencing. PCR products were subsequently verified and bands were visualized under UV. DNA purification was performed by the AMPure XT beads prior to quantification performed using Qubit, and equal amounts of DNA from each sample were pooled. DNA sequencing was performed on an Illumina Miseq instrument according to the Illumina Miseq. 16s Metagenomic Sequencing Library Preparation protocol (Illumina, San Diego, CA, USA).

### Sequence analysis

The quality of the raw sequence data was initially evaluated with FastQC (http://www.bioinformatics.babraham.ac.uk/projects/fastqc/). Consequently, de-multiplexing was performed to remove PhiX sequences. Individual sample sequence was assigned based on their dual-index barcodes, allowing for 1 mismatch by using custom Perl scripts. These selected, high quality sequences were further processed using Mothur, which was illustrated on the Mothur website (http://www.mothur.org/wiki/Miseq Sop)^23^. Paired end reads of sufficient length were firstly merged into full-length sequence (tag), removing either tags with high amount of ambiguous bases and homo-polymers, or tags out of expected range. Then, sequences were aligned to SILVA^24,25^.

16S rRNA gene sequences were selected according to the correct aligned region and were consistent with the same alignment coordinates^26^. For each sample, the frequency of the unique sequence was identified and a pre-clustering algorithm was utilized for further denoising^27^. The resulting sequences were then screened and the chimeric sequences were discarded based on prediction by UCHIME using the reference database mode^28^. Substantial taxonomic ranks were assigned to each sequence using Ribosomal Database Project (RDP) Naive Bayesian Classifier^29^ trained on the RDP 16S rRNA gene training set (version 10). Next, an 80% pseudo-bootstrap confidence score was required as a cutoff to achieve a balance between accuracy and the retained number of reads. Sequences that could not be classified at the kingdom level or that classified as Archaea, Eukaryota, chloroplasts, or mitochondria were culled. Finally, sequences were split into groups corresponding to their taxonomy at the level of order, and then were assigned to operational taxonomic units (OTUs) at 97% similarity level^27^.

### Statistical analysis

The statistical analysis was performed using the R software (version 3.3.3). Continuous variables were presented as mean ± standard deviation, while group comparisons were conducted by independent T test. Categorical variables were presented as count, and relevant group comparisons were performed using Chi-square test or Fisher Exact test. *P* < *0.05* was defined as statistically significant.

### Data availability

The datasets generated during and/or analysed during the current study are available from the corresponding author on request.
